# Reliability and Efficiency of the CAPRI-3 Metastatic Prostate Cancer Registry Driven by Artificial Intelligence

**DOI:** 10.3390/cancers15153808

**Published:** 2023-07-27

**Authors:** Dianne Bosch, Malou C. P. Kuppen, Metin Tascilar, Tineke J. Smilde, Peter F. A. Mulders, Carin A. Uyl-de Groot, Inge M. van Oort

**Affiliations:** 1 Department of Urology, Radboud University Medical Center, 6525 GA Nijmegen, The Netherlandsinge.vanoort@radboudumc.nl (I.M.v.O.); 2 Department of Radiotherapy, Maastro Clinic, 6229 ET Maastricht, The Netherlands; 3 Department of Medical Oncology, Isala Hospital, 8025 AB Zwolle, The Netherlands; 4 Department of Medical Oncology, Jeroen Bosch Hospital, 5223 GZ ‘s-Hertogenbosch, The Netherlands; t.smilde@jbz.nl; 5 Erasmus School of Health Policy and Management, Erasmus University Rotterdam, 3062 PA Rotterdam, The Netherlands

**Keywords:** prostate cancer, registry, text mining, artificial intelligence

## Abstract

**Simple Summary:**

CAPRI-3 is an observational registry on metastatic prostate cancer that uses artificial intelligence (AI) for patient identification and data collection. The aim of this study is to demonstrate the reliability and efficiency of this method. Our deliberate effort to maximize the negative predictive value of our patient-identification algorithm to rule out unsuitable candidates without manual screening was successful and reached 94.8%. Completeness and accuracy of data extraction were 92.3% or higher but were lower (up to 10%) for date fields and inaccessible data (images/pdf). The AI-driven approach, including additional manual quality control, was much faster than full manual data collection (105 vs. 300 min per patient). In conclusion, the AI-driven approach of the CAPRI-3 registry is largely reliable and timesaving but manual quality control is needed for the less reliable and inaccessible data.

**Abstract:**

Background: Manual data collection is still the gold standard for disease-specific patient registries. However, CAPRI-3 uses text mining (an artificial intelligence (AI) technology) for patient identification and data collection. The aim of this study is to demonstrate the reliability and efficiency of this AI-driven approach. Methods: CAPRI-3 is an observational retrospective multicenter cohort registry on metastatic prostate cancer. We tested the patient-identification algorithm and automated data extraction through manual validation of the same patients in two pilots in 2019 and 2022. Results: Pilot one identified 2030 patients and pilot two 9464 patients. The negative predictive value of the algorithm was maximized to prevent false exclusions and reached 94.8%. The completeness and accuracy of the automated data extraction were 92.3% or higher, except for date fields and inaccessible data (images/pdf) (10–88.9%). Additional manual quality control took over 3 h less time per patient than the original fully manual CAPRI registry (105 vs. 300 min). Conclusions: The CAPRI-3 patient-identification algorithm is a sound replacement for excluding ineligible candidates. The AI-driven data extraction is largely accurate and complete, but manual quality control is needed for less reliable and inaccessible data. Overall, the AI-driven approach of the CAPRI-3 registry is reliable and timesaving.

## 1. Introduction

The treatment landscape of metastatic hormone-sensitive prostate cancer (mHSPC) and castration-resistant prostate cancer (CRPC) significantly changed over the past decade from treatment with androgen deprivation therapy alone to the availability of multiple life-prolonging drugs (LPDs) such as taxane-based chemotherapy and novel hormonal agents. Although LPDs were first registered for CRPC only, lately the indication was extended to the (upfront) treatment of mHSPC as well [1,2,3].

LPDs are primarily analyzed in randomized clinical trials (RCTs). Although RCTs remain the gold standard, several limitations are recognized. RCTs, in general, are associated with patient selection (clinical trial participants are younger and healthier), lack of external validity, and publication bias [4,5]. Moreover in prostate cancer, there is a lack of comparative RCTs and RCTs on treatment sequencing [3]. Also, the effect of mHSPC treatments on the effectiveness of CRPC treatments is unknown since LPDs registered for CRPC were investigated in an era prior to mHSPC registration. Recent developments in novel next-generation imaging methods, biomarkers, and molecular characterization resulted in even more unanswered questions. Consequently, clinicians experience increasing difficulty in determining the optimal treatment for their patients [6,7,8,9].

Disease-specific patient registries can be a valuable addition to evidence gained in RCTs and can help fill the knowledge gap, as they gather real-world data of the population as a whole [5]. Furthermore, a registry is a relatively cost-effective tool in a rapidly changing treatment landscape [10]. A major shortcoming of disease registries is, however, that most clinical data are still collected manually and transcribed onto electronic registry databases. Such manual labor interferes with the quality of data due to manual errors and it is time-consuming and expensive [11].

An automated and reliable approach to a (metastatic prostate cancer) registry is necessary to efficiently provide up-to-date data to clinicians. This can be a challenge because patient data in electronic health records (EHRs) are predominantly stored as text. However, in recent years, a range of artificial intelligence (AI) technologies have emerged that enable the extraction of meaningful data from unstructured text fields [12]. This practice is commonly referred to as ‘text mining’ and includes several technologies, such as natural language processing (NLP) and named entity recognition (NER). In the Netherlands, specific text-mining software that uses NLP is available in a large quantity of hospitals, which enables researchers and clinicians to gather data from EHRs in a semiautomated manner [13,14].

From 2010 up to 2016, real-world data of 3600 CRPC patients in the Netherlands were manually collected in the original CAPRI registry, which led to important new insights in clinical daily practice [15,16,17,18,19,20,21]. Due to the rapid changes in the treatment landscape of metastatic prostate cancer and the urge to collect data in a more efficient manner, we initiated the AI-driven CAPRI-3 registry in 2021. In this registry, we collect data from mHSPC and CRPC patients by using text-mining software [13].

In this paper, we provide insight into the AI-driven (i.e., semiautomated) approach of the CAPRI-3 registry and evaluate the reliability and efficiency of the patient-identification and data-collection process.

## 2. Materials and Methods

CAPRI-3 is an observational retrospective multicenter cohort registry that monitors the real-life daily practice of patients diagnosed with metastatic HSPC and/or CRPC between 2016 and 2021. Metastatic HSPC is defined as the involvement of lymph nodes, bones, or viscera on radiological assessment or as defined by the treating doctor/physician (i.e., palliative treatment with surgical or medical castration). The definition of CRPC is prostate cancer that is progressing despite medical or surgical castration according to the European Urology Association (EAU) guideline of 2023 [22] or defined by the treating doctor/physician (i.e., recorded castration-resistance or prescription of a new anticancer treatment because of biochemical, radiological, or clinical progression despite surgical or medical castration).

### 2.1. Patient Identification

Patients diagnosed with mHSPC and/or CRPC between 2016 and 2021 are retrospectively included after written informed consent for accessing and processing data from their medical files. Necessary up-to-date information to contact patients who are lost to follow-up (lack of scheduled future appointments or appointments in the last 270 days with a urologist or medical oncologist) is not available in the Netherlands due to privacy legislation. Therefore, these patients and deceased patients are exempted from informed consent. 

To ensure the collection of representative real-world data, no exclusion criteria were formulated. If patients are lost to follow-up after a second opinion without treatment or after a single diagnostic procedure without treatment, they are merely excluded because of a lack of data. All patients that objected to the use of their medical data for scientific research in their EHRs are also excluded from the study. 

All of the patients are identified using the CTcue Patient Finder software (CTcue B.V., Amsterdam, The Netherlands), which enables users to search through the EHRs for eligible patients through text mining [13]. The software complies with the General Data Protection Regulation (GDPR) by giving users access to minimal datasets stripped of content that can be linked to the patients. With the support of CTcue B.V. experts in patient identification and data handling, we created a query to identify metastatic HSPC and CRPC patients based on their disease characteristics. The CAPRI-3 patient-identification query consists of 4 steps:

1. Identification of patients with a urology and/or internal medicine DBC code (in Dutch: Diagnose Behandeling Combinatie, a code used for reimbursement);
2. Identification of patients based on mHSPC- and CRPC-specific systemic treatments supplied by clinical pharmacies (i.e., cabazitaxel, abiraterone, enzalutamide, radium-223);
3. Identification of patients based on textual notification (including synonyms, abbreviations, and common typos) of “metastatic prostate cancer” or “castration-resistant prostate cancer” in the EHR;
4. Identification of patients based on chemical castration treatments supplied by clinical pharmacies (i.e., LHRH-agonists and -antagonists) or textual notification (including synonyms, abbreviations, and common typos) of chemical castration treatments in the EHR and/or conducted surgical castration.

In the first pilot in 2019, we created an algorithm in two medical centers (secondary and tertiary care with different EHR systems) to calculate the chance of inclusion. Elements of the query with a high predictive value were included in the algorithm to bulk patients for in- and exclusion (i.e., in- or exclude groups of patients based on these elements without manual screening). Based on the results of the first pilot, the algorithm was slightly modified in the second pilot to maximize the negative predictive value (NPV) and, thereby, prevent false exclusions. Furthermore, instead of excluding patients without any urology or internal medicine outpatient visits, at least one outpatient visit was added as an inclusion criterium. Both pilot algorithms were validated by checking all bulk in- and exclusions manually.

In the current practice of the CAPRI-3 registry, all bulk inclusions are still validated manually by trained datamanagers. However, based on the results of the second pilot, patients without predictive algorithm elements are not manually screened for eligibility for inclusion anymore.

### 2.2. Data Extraction and Storage

After patient identification, the data of included subjects are extracted by use of the CTcue Data Collector software (Version 4.7.0, CTcue B.V., Amsterdam, The Netherlands). This software enables users to search and extract structured data (e.g., lab results, surgical procedures, and imaging techniques) as well as unstructured data (i.e., text fields) in the EHRs through text mining [13]. 

Data of the included subjects are transported onto the Digital Research Environment of Radboud University Medical Center using Castor Electronic Data Capture (Version 2023.2.2.2, Castor Research Inc., Amsterdam, The Netherlands) [23]. Only indirectly identifying (i.e., coded) data are centrally stored in this web-based Case Report Form (eCRF). 

The completeness and accuracy of the automated data extraction were analyzed in the first pilot in 2019. Based on the results of this sample, less reliable data were extrapolated to all eCRF questions with the same type of data fields. We identified data fields needing manual validation (mainly unstructured data) and fields that can be extracted without manual validation (structured data). 

### 2.3. Quality Control

Quality control consists of all of the following:

5. Automatic checks are programmed into the eCRF to make sure data meet certain required formats and ranges to maximize accuracy;
6. Based on the results of the pilot, unreliable automatically extracted data fields are manually validated by trained datamanagers. The remaining unanswered eCRF questions are manually collected from the EHR. In particular, scanned forms and pdf files are examined, which are inaccessible to the software;
7. During the periodic quality checks, the coordinating researchers analyze the manually completed data for discrepancies and/or missing values. Any found (manual) errors are subsequently checked at the source and altered accordingly. All activities and alterations are logged in Castor [23].

For components 2 (manual data validation and completion) and 3 (quality checks of manually completed data), standard operating procedures (SOPs) are in place. 

### 2.4. Outcomes

The primary outcomes of this study are:

- Negative predictive value (NPV) of the patient-identification algorithm;
- Accuracy (i.e., percentage of data free from error) and completeness (i.e., percentage of the total amount of data) of the automated data extraction.

Secondary outcomes are sensitivity, specificity, and positive predictive value (PPV) of the patient-identification algorithm, overall time-effectiveness, and associated costs. 

## 3. Results

### 3.1. Patient Identification

**Figure 1:**
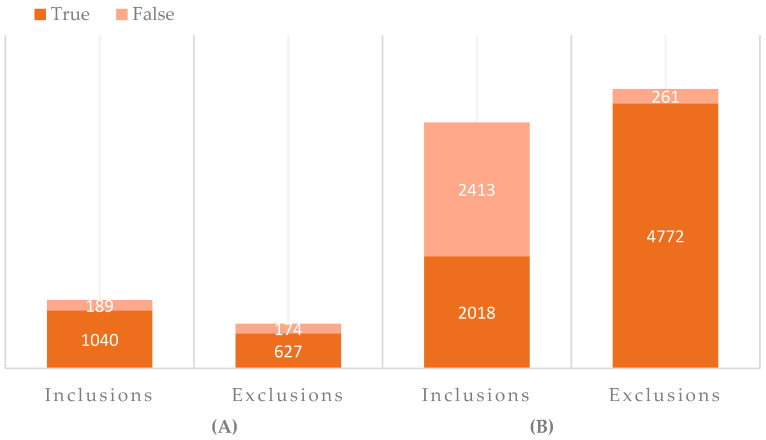
Manual validation of the patient-identification algorithms. (**A**) Amount of in- and exclusions in the first pilot in 2019; identified exclusions and remaining identified subjects by the algorithm are summed up as exclusions. (**B**) Amount of in- and exclusions in the second pilot in 2022.

In the first pilot, 2030 patients were identified by the algorithm, with 1229 inclusions, 452 exclusions, and 349 remaining subjects (Figure 1, Table 1). Overall, 189/1229 (15.4%) were false inclusions, 5/452 (1.1%) were false exclusions, and the 169/349 (48.4%) remaining subjects could still be included after manual screening. Since only 1.1% of exclusions by the pilot algorithm were false, bulk exclusions were eliminated from the second pilot algorithm altogether and replaced with additional inclusion criteria in the patient-identification query. 

In the second pilot, 9464 patients were identified by the algorithm, with 4431 inclusions and 5033 remaining subjects (Figure 1, Table 1). Overall, 2413/4431 (54.5%) were false inclusions and the 261/5033 (5.2%) remaining subjects could still be included after the manual screening. 

The reasons for false positivity were analyzed in a sample of 20 patients per hospital in the second pilot. Twenty-one patients (35%) were manually excluded due to the absence of metastatic disease, 20 patients (33.3%) due to lack of data (e.g., second opinions without follow-up), and 19 patients (31.7%) due to diagnosis dates (mHSPC and/or CRPC) outside the study period. 

In Table 2, the accuracies of both pilot algorithms are summarized. The negative predictive value (NPV) of the algorithm was maximized in the second pilot and reached 94.8%. After two pilots, the overall sensitivity was 88.5% and the specificity was 66.4%. 

### 3.2. Data Extraction

The extracted data showed completeness rates of 100% or higher (higher in case automated data extraction exceeded the amount of manually collected data), except for the Gleason score (94.4%) and drug treatments (84.4%) (Table 3). All missing data were inaccessible for the software (1/18 Gleason scores stored as a pdf and 5/32 patients treated elsewhere). For weight and hemoglobin, the software was able to extract more measurements than manual data collection (500% and 142.9%, respectively). On average, completeness was 146.9%. The accuracy of the automated data extraction compared to manual data collection of the same subjects was 92.3% or higher, except for the initial diagnosis date and start dates of drugs (10–60%). On average, the accuracy was 87.4%.

### 3.3. Quality Control

The average time needed for the manual screening of subjects without predictive algorithm elements by trained datamanagers in the second pilot was 2.7 min (162 s) per patient.

True inclusions accounted for 21.3% of the total amount of identified patients and predictive algorithm elements were absent in 53.2% of identified patients. The goal of the CAPRI-3 registry is set for 10,000 inclusions, which corresponds to 49,554 identified patients, of which 26,363 were without predictive algorithm elements (remaining subjects). Manual screening of these remaining subjects will take approximately 1186 h in total. In the Netherlands, 1872 working hours per calendar year (36 h per week) are considered a full-time healthcare job and corresponds to a minimum of 58,944 euro per year (4912 euro per month) on employee costs (based on the Dutch collective employment agreement of university medical centers for student-assistant jobs). Thus, 1186 h of manual screening of the remaining subjects for CAPRI-3 as a whole corresponds to 0.63 full-time-equivalent (FTE) of work or 37,344 euro.

The average time needed for datamanagers to manually validate and complete the extracted data showed a learning curve and was 105 min per patient on average. This consisted of 38 min (range 25–65) needed for manual data validation in the CTcue software and 67 min (range 28−93) in Castor. Compared to the precursor CAPRI registry, which took 300 min per patient to manually identify and collect data, the CAPRI-3 semiautomated approach saves approximately 195 min (65%) per patient. This adds up to a time savings of 32,500 h for 10,000 patients in total, which corresponds to 17.4 FTE of work or 1,023,333 euro (approximately 102 euro per patient).

## 4. Discussion

In this paper, we report the results of two pilots on the reliability and efficiency of the AI-driven CAPRI-3 registry on metastatic prostate cancer. This semiautomated approach involves using an algorithm to identify patients and text-mining software to extract data.

The patient-identification algorithm was optimized by elaborate testing in two pilots because mHSPC and CRPC patients are not easily identified. These diagnoses are not reported in a structural manner in the Dutch EHRs and they lack a common denominator for easy identification. After two pilots, the final CAPRI-3 algorithm is now well able to rule out ineligible candidates in an efficient manner: the algorithm reached an NPV of 94.8% and reduced the number of patients that needed to be screened by 53.2%. In a recent Dutch study that used the same software application, their algorithm missed 17.6% of actual trial participants (due to missing in- or exclusion criteria in these participants) and the number of patients who needed to be screened manually was reduced by 79.9% [24]. Studies that used different text-mining applications for patient identification showed reductions up to 92% and missed 5–10% of candidates (i.e., sensitivity or ‘recall’ rates of 90–95%) [25,26].

The PPV and specificity of our second pilot patient-identification algorithm (45.5 and 66.4%, respectively) were overall poor and (much) lower compared to our first algorithm. The primary reason for this is the deliberate effort to maximize the NPV. Additionally, through the elimination of bulk exclusions from the algorithm in the second pilot, rightful exclusions were taken out of the equation and, therefore, lowered the specificity. However, PPV and specificity of the patient-identification algorithm are of much less relevance for our registry because they do not impact efficiency or reliability. The reason for this is that automatically included subjects will be manually validated anyway. On the other hand, the maximized NPV rendered the need for additional manual validation of remaining subjects without predictive elements obsolete, improving time and cost efficiency. Instead, once every 6 months, these patients will be checked for newly acquired predictive algorithm elements.

Our data, in combination with current data in the literature, emphasize that screening of eligible subjects for research by use of text-mining software is feasible, time efficient, accurate, and can augment clinical trial enrollment. We used a specific type of text-mining software that is available in the Netherlands but there is a wide spread of other applications available elsewhere (an online search of ‘software for medical data extraction’ produces 12 million results). The Australian Government’s Practice Incentives Program (PIP) Quality Improvement (QI) Incentive even requires clinicians that want to participate to have a data-extraction tool installed [27].

As for the automated data extraction, our completeness and accuracy rates were overall very high: most data fields showed rates of 92.3% or higher, with an average accuracy of 87.4% and completeness of 146.9%. Nevertheless, low accuracy rates (up to 10%) were encountered in date fields (i.e., diagnosis dates and start dates of drugs) and completeness was compromised (up to 50%) by data that was inaccessible for the software we used (i.e., data stored as image/pdf and patients treated elsewhere).

We found two Dutch studies that used the same text-mining software application as we did. One showed a surprisingly similar average accuracy (i.e., ‘agreement’) rate of 87.1% that ranged between 62.6 and 99.6% per collected variable [24]. The other study only presented their performance scores as PPV (i.e., ‘precision’) and sensitivity (i.e., ‘recall’) rates per variable, which also showed wide ranges of 39.1–100 and 63.2–100%, respectively [14]. Their ranges are quite similar to ours, although not as pronounced, which can be explained by the absence of collected date fields that revealed the most compromised accuracies in our study. One of these studies also reported the availability of automatically extracted data compared to manually collected data (i.e., completeness) as a median absolute difference rate of 2.8% (IQR 0.4–8.5%), which is a little less favorable, but comparable, to our results [24]. Surprisingly, drug treatments were more complete in their automatically extracted data, possibly because manual data collection consisted of face-to-face interviews with participants rather than consultation of the EHRs, but this is not further addressed by the authors.

Furthermore, we identified several studies that used different software applications employing the same AI technology (NLP) for extracting data from large volumes of unstructured texts, such as clinic letters, mental health records, and radiology and colonoscopy reports [28,29,30,31,32]. The results of these studies are demonstrated in various ways but the accuracies they presented were overall very high and slightly better than ours (ranging between 86.1 and up to >95%). Once again, several researchers experienced large variations in accuracies between variables or models (compromised up to 57% or excluded due to ‘poor performance’), which they attributed to formatting and reporting variations and interpretation challenges. This highlights the need for manual and/or external validation of text-mining applications.

To ensure good quality despite lower accuracy rates for certain data fields, CAPRI-3 was designed with an extensive quality-control protocol that involves manual validation of automatically extracted data and subsequent quality checks for errors in the manually validated data. We compared the time needed for manual quality control in the semiautomated CAPRI-3 approach to the original CAPRI registry, which fully relied on manual identification and collection of data. The semiautomated approach was much faster (105 min vs. 300 min, almost a threefold reduction) and showed a learning curve. Earlier Dutch research showed that the software has the potential to quicken procedures even by sevenfold [14].

Despite such advances, it would be immensely beneficial if manual quality control became outdated. This can be achieved if patient data in EHRs are structurally reported in a categorical manner and/or through advancements in AI software, such as machine-learning (ML) and deep-learning (DL) technologies. ML is the ability of systems to learn from problem-specific data using algorithms without being explicitly programmed for the task, whereas DL is a specialized ML concept in which artificial neural networks are used (inspired by the human brain) [33,34]. These models play a critical role in the development of learning health systems that leverage big data (data too large or complex for traditional data-processing software applications) for research purposes [35]. CTcue B.V. is already working on integrating ML technology into their software applications [13] and DL models are rapidly emerging in various areas of oncology, such as histopathology, molecular subtyping, and cancer diagnosis [36,37]. Specifically, research into prostate cancer has demonstrated promising performances of ML models in detecting prostate cancer on MRI scans. Their best performing models achieved area under the curve (AUC) rates between 0.78 and 0.87 [38,39,40,41], surpassing the diagnostic performance of radiologists in one paper (AUC 0.81 vs. 0.69, *p* = 0.02) [39] and enhancing the specificity of PI-RADS assessment by senior radiologists in another (from 52.5% to 72.6%) [40].

In a recent systematic review on DL solutions for challenges in data representation in EHRs, the authors address that for DL algorithms to be successful, large-scale EHR datasets with high-quality data is necessary [42]. However, most research focuses on prediction modeling and data utilization, not on the extraction of data. As to our knowledge, there are currently only three published articles on DL models for EHR data extraction. Their results are very promising with accuracy rates of 91.3–95% and AUC values of more than 0.98 [43,44,45]. However, once more we must emphasize the need for generalizability and external validation, as the knowledge acquired by a model in one particular setting does not necessarily fit another setting. As demonstrated in a recent study on the external validity of an NLP algorithm for identifying stroke-related data from radiology reports, the performance score of the algorithm was affected by inconsistent report styles and vocabulary among radiologists [46].

This study recognizes a few limitations. First, although manual data collection is the gold standard, it is associated with manual errors. By means of quality control, we try to correct most of these errors but one can think of several examples in which they are undetected, such as errors in interpretation or errors in data entry within the ranges of the data field [47].

Second, we estimated that 20 subjects would be a representative sample size for the analysis of completeness and accuracy of the automatically extracted data. We did not further investigate whether the sample was representative. Nevertheless, the results are in line with the literature and our extensive experience in data collection with the CTcue software.

Lastly, we did not consider how much time (months) we invested in the creation of the software query and the patient-identification algorithm. For large or complex study populations, the benefits will most likely outweigh the costs; for small registries, this is probably not the case.

## 5. Conclusions

The CAPRI-3 patient-identification algorithm is a sound replacement for manual identification to rule out ineligible candidates. The automated data extraction is complete and accurate for most data fields. However, manual quality control is still necessary for less reliable and inaccessible data fields. Overall, the AI-driven approach to the CAPRI-3 registry is reliable and time efficient.

## Figures and Tables

**Table 1:** Manual validation of the patient-identification algorithms.

	Pilot 1 (2019)	Pilot 2 (2022)
Identified inclusions by algorithm, n (%)	1229 (60.5)	4431 (46.8)
True inclusions, n (valid %)	1040/1229 (84.6)	2018/4431 (45.5)
False inclusions, n (valid %)	189/1229 (15.4)	2413/4431 (54.5)
Identified exclusions by algorithm, n (%)	452 (22.3)	NR
True exclusions, n (valid %)	447/452 (98.9)	
False exclusions, n (valid %)	5/452 (1.1)	
Remaining identified subjects by algorithm, n (%)	349 (17.2)	5033 (53.2)
True exclusions, n (valid %)	180/349 (51.6)	4772/5033 (94.8)
False exclusions, n (valid %)	169/349 (48.4)	261/5033 (5.2)

Abbreviations: NR, no result.

**Table 2:** Accuracies of the patient-identification algorithms.

	Pilot 1 (2019)	Pilot 2 (2022)
Sensitivity	85.7%	88.5%
Specificity	76.8%	66.4%
Positive predictive value (PPV)	84.6%	45.5%
Negative predictive value (NPV)	78.3%	94.8%
Accuracy	82.1%	71.7%

**Table 3:** Completeness and accuracy of the automated data extraction compared to manual data collection of the same subjects.

	Manually n = 20	Automated n = 20	Completeness	Accuracy
Date of initial diagnosis, n (%)	20/20 (100)	20/20 (100)	20/20 (100)	2/20 (10)
				20/20 (100) ^A^
Type of tumor, n (%)	18/20 (90)	18/20 (90)	18/18 (100)	18/18 (100)
Adenocarcinoma	18/20 (90)	18/20 (90)		
Unknown	2/20 (10)	2/20 (10)		
Gleason score, n (%)	18/20 (90)	17/20 (85)	17/18 (94.4) ^B^	16/17 (94.1) ^C^
6–7	10/20 (50)	8/20 (40)		17/17 (100) ^A^
8–10	8/20 (40)	9/20 (45)		
Unknown	2/20 (10)	3/20 (15)		
Weight, n (%)	1/20 (10)	5/20 (25)	5/1 (500)	5/5 (100)
ECOG PS, n (%)	0/20 (0)	1/20 (5)	1/1 (100)	-
PSA, n (%)	20/20 (100)	17/20 (85)	17/20 (85)	17/17 (100)
		20/20 (100) ^D^	20/20 (100) ^D^	
Hb, n (%)	14/20 (70)	13/20 (65)	13/14 (92.9)	13/13 (100)
		20/20 (100) ^D^	20/14 (142.9) ^D^	
MDT-date, n (%)	NR	13/20 (65)	-	12/13 (92.3)
Drug treatment, n (%)				
Docetaxel	8/20 (40)	6/20 (30)	6/8 (75)	6/6 (100)
Abiraterone	14/20 (70)	13/20 (65)	13/14 (92.9)	13/13 (100)
Enzalutamide	6/20 (30)	5/20 (25)	5/6 (83.3)	5/5 (100)
Cabazitaxel	2/20 (10)	1/20 (5)	1/2 (50)	1/1 (100)
Radium-223	2/20 (10)	2/20 (10)	2/2 (100)	2/2 (100)
Total			27/32 (84.4) ^E^	27/27 (100)
Start date of drug, n (valid %)				
Docetaxel	NR	6/20 (30)	6/6 (100)	6/6 (100)
Abiraterone	NR	13/20 (65)	13/13 (100)	12/13 (92.3) ^F^
Enzalutamide	NR	5/20 (25)	5/5 (100)	3/5 (60) ^G^
Cabazitaxel	NR	1/20 (5)	1/1 (100)	1/1 (100)
Radium-223	NR	2/20 (10)	2/2 (100)	2/2 (100)
Total			27/27 (100)	24/27 (88.9)
Dose of drug, n (valid %)				
Docetaxel	NR	0/20 (0)	0/NR (NR)	-
Abiraterone	NR	13/20 (65)	13/NR (NR)	13/13 (100)
Enzalutamide	NR	5/20 (25)	5/NR (NR)	5/5 (100)
Cabazitaxel	NR	0/20 (0)	0/NR (NR)	-
Radium-223	NR	0/20 (0)	2/NR (NR)	2/2 (100) ^D^
Total			20/NR (NR)	20/20 (100)

Abbreviations: CAPRI, Castration-Resistant Prostate Cancer Registry; ECOG, Eastern Cooperative Oncology Group; PSA, Prostate Specific Antigen; Hb, Hemoglobin; MDT, Multidisciplinary team; NR, no result. ^A^ Accuracy after manual validation (i.e., quality control). ^B^ N = 1 Inaccessible data (pdf file). ^C^ N = 2 Upgrading of manual data collection (control group). ^D^ When searched in unstructured text fields. ^E^ N = 5 Inaccessible data (treated elsewhere). ^F^ N = 1 Abiraterone treatment in trial. ^G^ N = 2 Inaccessible data (treated elsewhere).

## Data Availability

Restrictions apply to the availability of these data. Data were obtained from participating hospitals and are only available from the authors with the permission of these hospitals.
